# Improvement of life quality measured by Lansky Score after enzymatic replacement therapy in children with Gaucher disease type 1

**DOI:** 10.1002/mgg3.339

**Published:** 2017-10-25

**Authors:** Magdalena Cerón‐Rodríguez, Edgar Barajas‐Colón, Lyuva Ramírez‐Devars, Claudia Gutiérrez‐Camacho, Juan L. Salgado‐Loza

**Affiliations:** ^1^ Department of Lysosomal Diseases Hospital Infantil de México Federico Gómez Ciudad de México México; ^2^ Department of Education Hospital Infantil de México Federico Gómez Ciudad de México México; ^3^ SICODIC Ciudad de México México

**Keywords:** Children, enzymatic replacement therapy, Gaucher disease, imiglucerase, Lansky Score, quality of life

## Abstract

**Background:**

Gaucher disease type 1 (GD1, OMIM# 230800), is a condition with high impact in patient's quality of life (QoL). We report the improvement in QoL of children with GD1 measured by Lansky play‐performance scale (LS) after enzymatic replacement therapy (ERT) and to describe our experience in the treatment of children with GD1.

**Methods:**

Five children with diagnosis of GD1 received imiglucerase 60 mg/kg every two weeks. LS, hepatomegaly, splenomegaly, hemoglobin, platelets, and growth rate were measured every 6 months after beginning ERT for 30 months.

**Results:**

After ERT, LS increased significantly from 28 ± 16.48 points before ERT to 70 ± 10 (*P* = 0.0046) and 95 ± 10 (*P* = 0.0022) points after 6 and 30 months of ERT, respectively; hemoglobin and platelets changed significantly from 9.28 ± 0.61 to 12.40 ± 0.85 (*P* = 0.0198) and from 71.50 ± 14.89 to 205.00 ± 65.34 (*P* = 0.0428) after 30 months of ERT, respectively. All patients demonstrated decreased hepatic and splenic size with mean reductions of 66% and 80% at 30 months of treatment and the USG longitudinal axis was reduced in both liver and spleen after ERT.

**Conclusion:**

The use of ERT with imiglucerase 60 mg/kg every two weeks has substantial benefits and significantly improves QoL, assessed with Lansky Score, of the five children with GD1 studied.

## Introduction

Gaucher disease type I (GD1, OMIM# 230800), is a multi‐systemic metabolic disorder due to the inherited deficiency of the lysosomal enzyme β‐glucocerebrosidase (acid β‐glucosidase; D‐glucosyl‐N‐acylsphingosine glucohydrolase, GCase; EC 3.2.1.45) encoded by the human *GBA1* gene (GenBank reference sequence NG_009783.1) resulting in accumulation of glucosylceramide, also called glucocerebroside, within the lysosomes of cells, particularly macrophages, with damage to hematological, visceral, and bone tissues (Weinreb et al. 2013).

WHO defines Quality of life (QoL) as an individual's perception of their position in life in the context of the culture and value systems in which they live and in relation to their goals, expectations, standards, and concerns (The WHOQOL Group, 1994). Patients with GD1 could range from asymptomatic subjects to those who display child‐onset disease affecting QoL, because the disease limits the goals and expectations, and affects the position in life of children and parents. Then, if we measure the change in the perception that children could have of their life we can assess the change in their QoL, because after treatment they could have an almost normal life instead of an ill‐related life. Damiano et al. (1998); Masek et al. (1999); Giraldo et al. (2000); Pastores et al. (2003); Giraldo et al. (2005); Weinreb et al. (2007); Oliveira et al. (2013) described the effect of enzymatic replacement therapy (ERT) on the QoL of adult patients with GD measured with SF‐36 score that is useful in patients older than 16 years of age. No studies in the literature have evaluated QoL in children with GD1. Lansky play‐performance scale for pediatric patients (LS) evaluates performance status using play activity, which is the main children activity, as index of QoL improvement in ambulatory or hospitalized children <16 years old.

The objective of this study was to report the change in QoL of children with GD1 measured by LS after ERT and to describe our experience in the treatment of children with GD1.

## Methods

Ethical compliance: this study was approved by ethics committee. All children with confirmed diagnosis of GD1 since May 2012 until October 2014 were admitted in the Lysosomal Disease Department of the Hospital Infantil de México Federico Gómez, to receive ERT every two weeks as outpatients. Initial GD1 severity was defined using the Zimran severity score index (ZS), higher ZS indicate greater clinical severity (≥20 severe; 11–19 moderate; 10–0 mild) (Zimran et al. 1992). All of them were diagnosed with determination of GCase activity and their genetic variation type was established by DNA isolation, polymerase chain reaction amplification of coding exons and sequencing with standard fluorescent sequencing protocol in both forward and reverse direction of amplifications to find disease causing‐variations in *GBA1* gen. (GenBank reference sequence NG_009783.1). After diagnosis, all received enzymatic replacement therapy with imiglucerase 60 mg/k every two weeks. The parents of the patients were informed about the study and an informed consent was obtained.

QoL was measured using Lansky play‐performance scale for pediatric patients (LS) (Lansky et al. 1987), that is a useful and easy to apply score for children age 1–16 years, hospitalized or not, for long term follow‐up or patients with active treatment. Originally, it has been used in patients with any type of malignancy and this is the first time that we use it for children with GD1. It was rated by parents based on their child's activity over the past week, keeping in mind good and bad days and an average of this period was rated comparing the description with the child reality. Level of activity was described in terms of active play, quiet play, degree of physical limitation, and degree of independence. Parents filled out the assessment based on the instructions on the form, and it was evaluated over time to assess for changes in performance status. The evaluation was ranging from fully active, normal (score, 100 points) to moderately restricted (score, 50–60 points), to completely disabled (score, 10 points or fewer).

Growth rate was defined with WHO or CDC growth charts in children under 2 years or older respectively, measuring height/length and weight to determine percentiles for length for age (LAP), weight for age (WAP) and length for weight (LWP). Children below third percentile were considered with low‐growth percentile.

Chitotriosidase serum level (EC 3.2.1.14) was measured with fluorometric enzymatic activity assay using 22μmolar 4‐methylumbelliferyl‐β‐D‐N, N′, N″‐triacetyl‐chitotrioside (4MU‐C3) as substrate (Turner 450 with 415 emission and 360 excitation filters, Laboratorio de Diagnóstico Bioquímico de Enfermedades por Depósito Lisosomal) and values between 28.1228 and 251.2198 nmol/mL/h were considered normal. Biomarkers hemoglobin (Hb), hematocrit (Ht) and platelets were measured before and after the treatment.

The hepatic and splenic edges were clinically and ultrasonographically evaluated. Hepatomegaly was considered when the hepatic edge was 2 cm or more below the right costal margin until 6 years old or if the edge was palpable after this age and splenomegaly was considered when the spleen edge was palpable below the left costal margin. Ultrasonographical liver and spleen enlargement was considered when the measures were larger than the values considered normal for the age and height.

LS, LAP, WAP, LWP, Hb, Ht, platelets, and clinical evaluation to measure hepatic and splenic edges were measured and registered before starting enzymatic replacement therapy (ERT) and in months 6, 12, 18, 24, and 30 after the beginning of ERT. Ultrasonogram (USG) to obtain liver and spleen longitudinal axis was performed at the beginning of ERT and every year after this measure. Chitotriosidase was measured only when was available due to budget constraints.

Positive treatment response was considered when after 12–24 months of ERT: Hb was maintained stable (>11 g/dL in girls or >12 g/dL in boys); platelets were increased 1.5–2 times the pretreatment level; hepatic and splenic volumes were reduced 20–30% and 30–40% respectively, chitotriosidase was reduced 40–70% and LAP, WAP and LWP were at least within percentiles 3 and 97 for corresponding sex and age.

Descriptive analysis with mean, standard deviation and percentages were calculated for continuous variables. Statistical analysis using GNU‐PSPP V3 statistical package was made with two‐tail paired *student t* when possible and significance was considered with *P* value ≤ 0.05.

## Results

**Figure 1:**
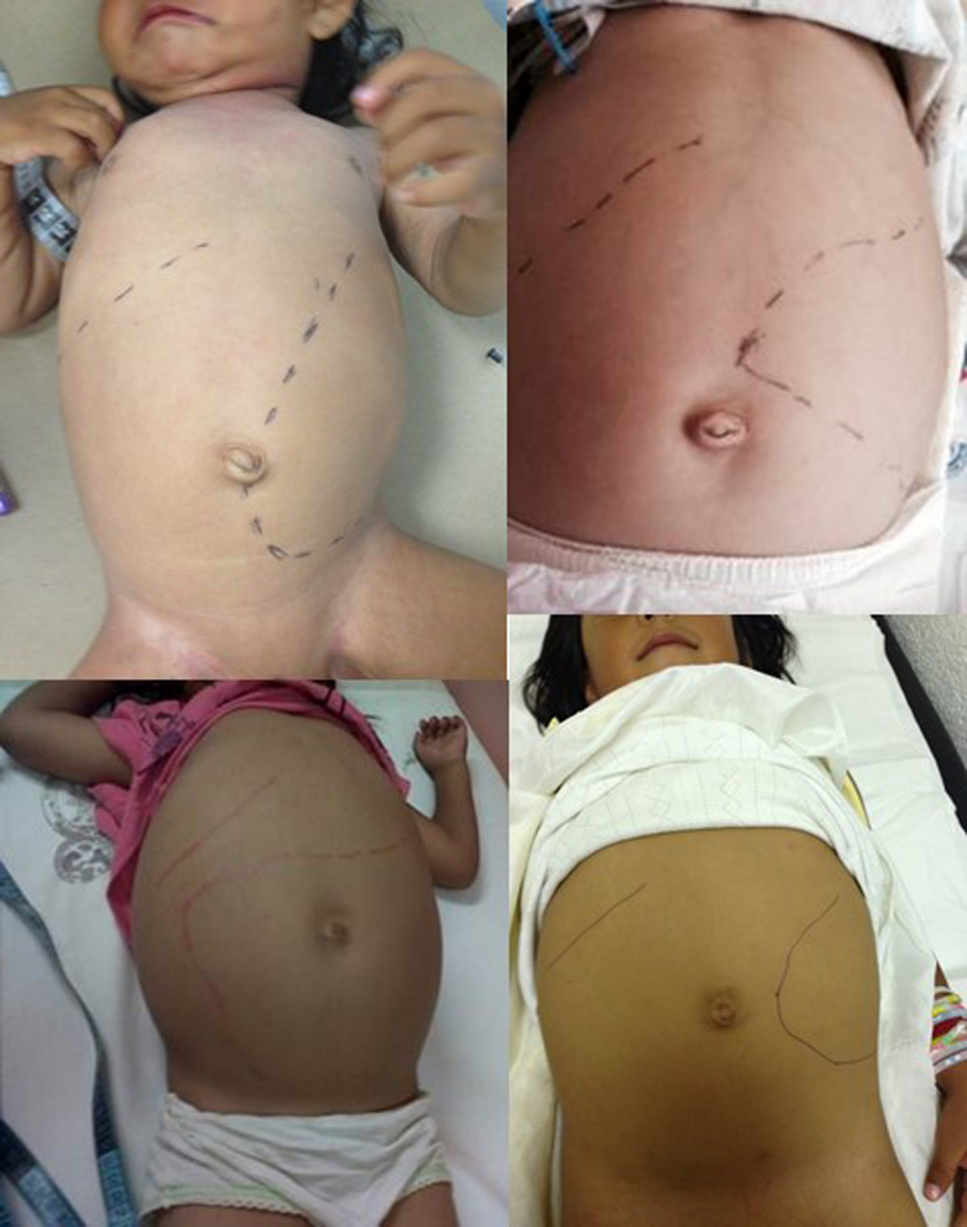
Patients 1–4 with hepatosplenomegaly before ERT.

Between May 2012 and October 2014, five children with GD1 were admitted in the Department of Lysosomal Diseases to receive ERT every two weeks as outpatients. The basal demographic and clinical characteristics are shown in Table 1. Three patients were male and two females. The severity of GD1 was considered mild in two and moderate in three by ZS. Three patients were younger than 2 years of age, one was 4 years and one almost 10 years of age at the moment of their admission. All of them had low GCase activity (Fig. 1). All patients, after treatment, reached clinical and laboratorial parameters to consider a positive response to ERT.

**Table 1:** Demographic characteristics of children with GD1 before ERT

Patient	1	2	3	4	5
Age (months)	18	50	12	15	117
Gender	Male	Female	Female	Male	Male
Ethnicity	Latin	Latin	Latin	Latin	Latin
Starting ERT dose (mg/day)	576	978	374	450	1638
Height (cm)	80	90	67	76	134
Weight (kg)	9.6	16.3	6.23	7.5	27.3
Zimram Severity Score	9	12	11	16	9
LAP (%)	12.3	0.2	0.3	0	30.1
WAP (%)	9.2	47.7	0.2	0.2	21.2
LWP (%)	15.1	99.7	1.5	7.5	20.3
Hb (g/dL) (nl 12–18)	10	9.5	9	8.6	12.3
Ht (mm) (nl 36–62)	30	30	29.4	27.2	36.4
Platelets (10^9^/L) (nl 145–450)	70	51	82	83	175
Hepatic edge (cm)	7	7	7	7	np
Hepatic longitudinal axis (cm)	11.8	14.7	12.9	12.8	13.2
Splenic edge (cm)	4	17	13	10	np
Splenic longitudinal axis (cm)	11.2	23.3	18.7	16.3	15.4
Lansky Score	40	20	10	20	50
GCase activity (μmol/mL blood/h) (nl > 3.61)	0.6	1.045	0.04	0.78	1.5
Chitotriosidase (nmol/mL plasma/h) (nl 28–251)	ND	ND	ND	7111.67	ND
Zygocity	Heterozygous	Homozygous	Homozygous	Homozygous	Heterozygous
Gen variation	Duplication p.Leu29Alafs*18/c.84dupG dbSNP 387906315 Substitution p.S356F/c.1184C>T	Substitution p.Leu483Pro/ c.1448T>C dbSNP 421016	Substitution Exon 10 p.Leu483Pro/c.1448T>C dbSNP 421016	Substitution Exon 11 p.Leu483Pro/c.1448T>C dbSNP 421016	Substitution Exon 11 p.Arg535His/c.1604G>A dbSNP 75822236 Substitution Exon 5 p.Met162Thr/c.485T>C dbSNP 794727783

LAP, length/height for age percentile; WAP, weight for age percentile; LWP, length/height for weight percentile; Hb, hemoglobin; Ht, hematocrit; np, not palpable; ND, not determined; nl, normal; GCase, β‐glucocerebrosidase.

LS were low (50 or less) in all cases at admission and the performance status was classified with moderate to severe limitations of physical activity and independence. LS increased significantly in all patients after ERT from a mean ± standard deviation of 28 ± 16.48 points before ERT to 70 ± 10 (*P* = 0.0046) after 6 months, 92.5 ± 9.57 after 12 months and 95 ± 10 (*P* = 0.0022) after 18 months and reached a plateau for the next evaluated periods receiving ERT. All changes in interim periods were not significant (Table 2).

**Table 2:** Changes in Lansky Severity Score before ERT and after 6, 12, 18, 24, and 30 months receiving ERT. All measures are points

Patient	Pre Tx	Months with ERT				
		6	12	18	24	30
1	40	60	100	100	100	100
2	20	80	90	100	100	100
3	10	60				
4	20	70	80	80	80	80
5	50	80	100	100	100	100
Mean ± SD	28 ± 16.43	70 ± 10^a^	92.5 ± 9.57^a^	95 ± 10^a^	95 ± 10^a^	95 ± 10^a^

a *P* < 0.05 versus values before ERT.

In four cases, Hb, Ht, and platelets were below the normal lower limit and in one patient, who was diagnosed at the age of 87 months and received cycles of ERT without a regular two weeks schedule during 30 months, the levels were normal. This patient was admitted in our hospital at the age of 117 months, when we start regular ERT cycles every two weeks and clinical/paraclinical basal data corresponds to this date. After ERT, in three patients, Hb, Ht, and platelets started to rise after 6 months of treatment and eventually reached normal levels. The patient with normal values, when was included remained with normal levels and one female patient died due to accidental choking after 6 months of ERT but her hematological parameters were below the normal limits in this period (Table 3).

**Table 3:** Changes in Hb and platelets before ERT and after 6, 12, 18, 24, and 30 months receiving ERT

	Before ERT	Months with ERT				
		6	12	18	24	30
Hb	9.28 ± 0.61	11.05 ± 1.42^a^	11.13 ± 1.26^a^	11.73 ± 1^a^	12.0 ± 0.89^a^	12.40 ± 0.85^a^
Platelets	71.50 ± 14.89	117.25 ± 44.51	125.00 ± 65.51	186.00 ± 37.36^a^	180.67 ± 51.73^a^	205.00 ± 65.34^a^

a *P* < 0.05 versus values before ERT.

**Figure 2:**
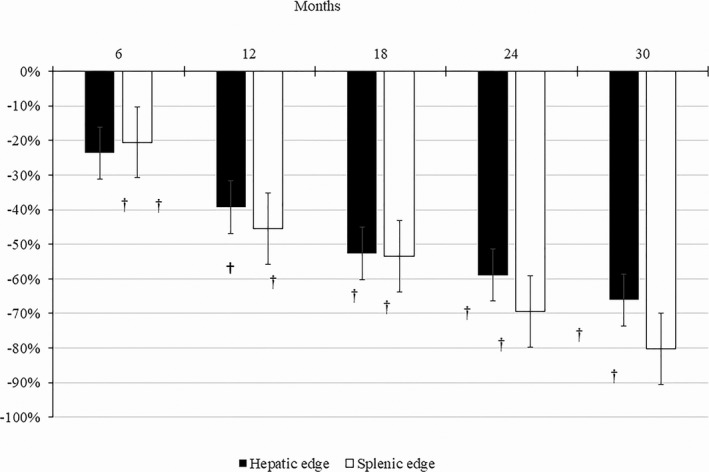
Mean percentage changes in hepatomegaly and splenomegaly after 6, 12, 18, 24, and 30 months of ERT. ^†^ *P* < 0.05 versus values before ERT.

**Figure 3:**
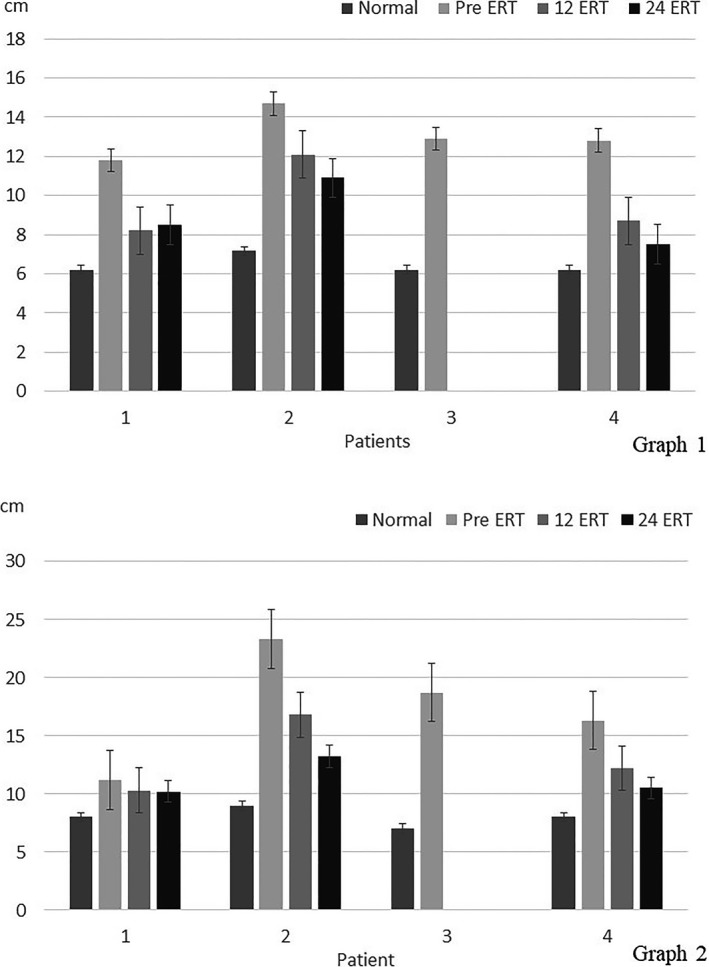
Values of USG longitudinal axis of liver (graph 1) and spleen (graph 2) before ERT and after 12 and 24 months of treatment.

Growth detention was referred by parents in all patients before ERT and low growth percentiles were considered in three patients, one for low LAP and two for low LAP and WAP, and were considered normal in two. All patients recover growth rate after ERT. Clinical and USG hepatomegaly and splenomegaly were found in four patients. One patient had liver and spleen between normal size parameters, but he received ERT before the organs had been measured. After ERT all patients decreased hepatic and splenic size with mean reductions of 66% and 80% at 30 months of treatment, respectively (Fig. 2). USG hepatic longitudinal axis was increased 50.5% over height adjusted normal length and splenic longitudinal axis 50.9% over age adjusted normal length. The hepatic longitudinal axis was reduced significantly in a mean of 26.7% (*P* = 0.016) and 31.7% (*P* = 0.020) and the splenic longitudinal axis was reduced in a mean of 20.4% (*P* = 0.142) and 29.3% (*P* = 0.165) after 12 and 24 months in all patients, respectively (Fig. 3).

Clinical neurological evaluation was performed and reported with normal psychomotor development and absence of meningeal, cerebellar, and neurocutaneous signs in all patients. All patients had normal computerized tomography and their magnetic resonance showed normal morphology of basal nuclei, cerebellum, brain steam and relation between cerebral gray and white matter. Ophthalmological exam reported normal pupillary reflexes, eye fund and visual acuity in both eyes; ocular movements were in orthoposition without saccadic, limitation or deviation movements. Electroencephalogram was described as normal without convulsive activity. Auditive evoked potentials were reported as normal in both ears.

Chitotriosidase was determined in four patients, in one before the beginning of the ERT, in two after 6 months of ERT and in one after two years of ERT. The level was over the normal high range in all patients. In one, chitotriosidase was never measured. In subsequent determinations, the levels tended to decrease in all patients but only two reached normal ranges.

## Discussion

GD1, has a wide heterogeneity of manifestation, it could be a severe, chronic, degenerative and sometimes life‐threatening condition with high impact in QoL mainly due to organomegaly, fatigue, bone pain, shortness of breath and bleeding tendency or be an asymptomatic disease. ERT can correct many of the pathophysiological disturbances and reverses the symptoms, improving patients QoL (Van Dussen et al. 2014). In previous studies, the effect of ERT in QoL of patients with GD1 measured with SF‐36 score was described but reports exclusively in children are not available and the evaluation of QoL with LS was never reported in this kind of patients. Damiano found an improvement in health‐related QoL of patients above 14‐year‐old with GD1 after ERT. In our study, we found a significant and increasing improvement every 6 months after ERT in LS of our patients that is consistent with Damiano′s enhance in the likely to report improvement in QoL measured with SF36 Health Survey when the time receiving ERT was longer (Damiano et al. 1998). Damiano also reported that QoL changes in younger GD patients was better than in older but both changes reached lower SF36 scores than the scores obtained in general population (Damiano et al. 1998) this situation was different for our patients because they reach LS scores considered normal after ERT perhaps due to the age at them start treatment and the lack of progressive disease damage. Masek informed a significant improvement from patient′s perspective in mood and global functioning and fewer psychological symptoms by adults with GD1 after 24 months of therapy (Masek et al. 1999) and Giraldo reported a statistically significant improvement of QoL in 35 patients with GD1 after the use of ERT using SF‐36 but not describes the age of the patients studied (Giraldo et al. 2000) and an improvement in self‐perception of global health ranging from 34.3% before ERT to 91.4% after ERT (*P* = 0.001) in patients older than 18 years (Giraldo et al. 2005). In both cases, the patient′s perspective improved after ERT; in the same way, our patients were significantly better in parent′s perspective when they saw their children doing more playing activities, because their symptoms were less than before ERT.

SF‐36 is a multidimensional score that assesses physical, emotional, mental health and other issues but is useful only in people older than 16 years, because the source of information is the patient who needs to discriminate between different options about their health status when completing the self‐applied questionnaire. On the other hand, LS is an easy QoL measuring tool, it can be applied as many times as needed, is useful in children between 1–16 years old and can evaluate improvement in patient's life. Perhaps, this score is only perceptual and in appearance only reflects the child′s parents observation of playing, this is the main child activity and can be limited by disease, affecting the degree of patient′s physical limitation and independence. Then, when the treatment achieves a positive response, it is possible to measure the changes due to less physical limitation and greater independence, reflecting an improvement in GD1 children QoL.

Other QoL questionnaires as PedsQL, that includes more dimensions, are available but due to our children′s parents educational limitations the risk of biased answers is high and misinterpretation of results could happen.

Like in adults, our studied children had statistically significant positive changes in QoL after ERT when measured with LS after six months of treatment and the improvement continued until the achieved scores were considered fully active. Two differences vs. adults or children older than 12 years can be seen, first the improvement was faster than in adults and second, children can reach scores considered normal. These changes in QoL were coincident with clinical modifications, sometimes statistically significant, in hepatosplenomegaly, Hb, platelets and improvement of growth percentiles. Although, in our study it was not possible to establish a relation between the use of ERT and the magnitude of treatment response, Oliveira reported that patients older than 12 years with GD receiving ERT had a clinically significantly better QoL than patients not receiving ERT (Oliveira et al. 2013), so that, like in other publications, we found a positive response to ERT that was associated to an significant improvement in QoL.

Our study is the first report of the changes in QoL of children with GD1 exclusively, because other reports uses adults or mixed populations that includes patients older than twelve years; also, describes the demographic characteristics and treatment responses of five Mexican children with GD1. We conclude that the use of ERT with imiglucerase 60 mg/kg every two weeks has substantial benefits and significantly improved QoL of the five children with GD1 studied, because it induces reduction in liver and spleen size, improves Hb and platelets and allows a near to normal musculoskeletal development, which had a positive impact in children activity performance.

## Conflict of Interest

Magdalena Cerón‐Rodríguez received reimbursement for attending a symposium and a fee for speaking from Sanofi Genzyme. Juan L Salgado‐Loza received payment for writing assistance from Sanofi Genzyme. Edgar Barajas‐Colón, Lyuva Ramírez‐Devars and Claudia Gutiérrez‐Camacho do not have competing interest. The authors confirm independence from the sponsors; the content of the article has not been influenced by the sponsors.
